# BNT162b2 COVID-19 vaccination uptake, safety, effectiveness, and waning in children and young people aged 5–11 years in Scotland

**DOI:** 10.7189/jogh.15.04250

**Published:** 2025-08-29

**Authors:** Igor Rudan, Steven Kerr, Christopher Sullivan, Karen Jeffrey, Zoe Grange, Lynda Fenton, Amanj Kurdi, Ting Shi, Lucy Cullen, Colin R Simpson, Srinivasa Vittal Katikireddi, Lewis D Ritchie, Chris Robertson, Aziz Sheikh

**Affiliations:** 1 Usher Institute, The University of Edinburgh, Edinburgh, UK; 2 Nuffield Department of Primary Care Health Sciences, Oxford University, UK; 3 Public Health Scotland, Glasgow, UK; 4 Strathclyde Institute of Pharmacy and Biomedical Sciences, Strathclyde University, Glasgow, UK; 5 College of Pharmacy, Hawler Medical University, Erbil, Kurdistan Region, Iraq; 6 College of Pharmacy, Al-Kitab University, Kirkuk, Iraq; 7 Department of Public Health Pharmacy and Management, School of Pharmacy, Sefako Makgatho Health Sciences University, Pretoria, South Africa; 8 School of Health, Wellington Faculty of Health, Victoria University of Wellington, Wellington, New Zealand; 9 MRC/CSO Social & Public Health Sciences Unit, University of Glasgow, Glasgow, UK; 10 Academic Primary Care, University of Aberdeen, Aberdeen, UK; 11 Department of Mathematics and Statistics, University of Strathclyde, Glasgow, UK

## Abstract

**Background:**

Few large-scale population studies have examined both safety and vaccine effectiveness (VE) specifically for 5–11-year-olds during the Omicron-dominant period in a setting with low vaccine uptake. The BNT162b2 (Pfizer-BioNTech) vaccine, administered in two doses, has shown strong efficacy against symptomatic and severe COVID-19 in clinical trials involving children and young people (CYP). Accordingly, we examined the uptake, real-world safety, VE, and waning of BNT162b2 VE against symptomatic COVID-19 among children aged 5–11 years in Scotland.

**Methods:**

This national prospective cohort study used the Scotland-wide Early Pandemic Evaluation and Enhanced Surveillance of COVID-19 (EAVE II) platform. We evaluated vaccine uptake using national data from the Turas Vaccination Management Tool, up to 16 April 2024. We assessed vaccine safety through national records on hospital admissions, employing a self-controlled case series design to examine 17 predefined health outcomes. We estimated VE against symptomatic, COVID-19 infection confirmed by reverse transcription-polymerase chain reaction and caused by the Omicron variant using a test-negative design.

**Results:**

From 19 March 2022 to 1 January 2023, 25.3% of the 392 658 children aged 5–11 years received their first COVID-19 vaccine dose and 16.2% completed the second dose. We found no increased risk of safety related hospital admission for 17 health outcomes in the post-vaccination period. During the Omicron period, VE against symptomatic COVID-19 was 60.8% (95% confidence interval (CI) = 0.3–84.5%) at 2–26 weeks post-first dose and 41.6% (95% CI = −89.6, 82.0) at 2–26 weeks post-second dose. The protective effect against symptomatic disease was no longer detectable in the period ≥27 weeks following both the first and second doses.

**Conclusions:**

The BNT162b2 vaccine demonstrated a strong safety profile in this age group. Receiving both doses was linked to a reduction in the risk of symptomatic COVID-19 during the Omicron variant period, but this protection waned over the following six months.

Early research into SARS-CoV-2 infection and COVID-19 in children and young people (CYP) primarily focused on clinical presentation, transmission patterns, viral load, diagnostic approaches, treatment strategies, and the debates surrounding vaccination in this age group [1,2]. Background information on vaccination of CYP to prevent COVID-19 and the related research on vaccine effectiveness (VE) and safety conducted to date is presented in **Box 1** [3–39]. While CYP generally experienced milder symptoms, a policy priority during the pandemic was protecting particularly vulnerable children [1]. Following the licensing of the first vaccines for adults, a complex debate emerged regarding the vaccination of CYP. The low risk of severe outcomes, as manifested by hospitalisations and deaths in this population, made discussions on vaccination both nuanced and, at times, polarising [2], as low risk of severe disease, unknown long-term consequences, and risk of infecting others in the household were weighted against potential side effects and related ethical issues when vaccinating low-risk children. This focused research efforts on three problems: determining ‘real-world’ VE in children aged 5–11 years during the Omicron period, which was relevant for ‘immune escape’; on the safety profile of BNT162b2 in a population at low risk of severe disease; and on immunity waning and its implications for vaccination policy.

Box 1 **Background information on vaccination of CYP to prevent COVID-19 and the related research on VE and safety conducted to date.**

Following the first clinical trials, the arguments in favour of vaccination of CYP were the excellent initial results on safety, immunogenicity, and efficacy of the two-dose BNT162b2 vaccine against COVID-19 disease and-or symptomatic infection in CYP. They have been initially demonstrated by the phase 3 clinical trials sponsored by the manufacturers – first those aged 16-17, then 12-15, and then 5-11 years [3–7].

Since then, tracking the uptake, monitoring safety, and analysing the VE and waning in the ‘real world’ context have emerged as policy priorities [8,9]. The first reports were published by research groups from China, Israel, the USA, and the UK (for England only), where the uptake of the first dose among the 12–17-year-olds was typically above 50%, while it was slightly lower for the second dose and in younger age groups [10–12]. The safety record was excellent to date, with rare myocarditis in boys aged 12–18 years following mainly the second dose of the BNT162b2 vaccine and sleep irregularities/disturbances being consistently reported as the main serious adverse effects among CYP [13–18]. The absolute risk for myocarditis and/or pericarditis was in the range of 1–5 per 100 000, being higher in second-dose recipients, and the risk of serious adverse effects following the third (booster) dose between 23 per 100 000 [19,20]. Among 5–11-year-olds, the reported risk of serious post-vaccination adverse effects following two doses is even smaller, about 12 per 100 000 [21].

The effectiveness of the first dose against symptomatic COVID-19 disease for the Delta variant in the 12–17 years age group typically ranged between 55–65%, and of the second dose between 87–99% [19,22–25]. The VE against more severe outcomes that required hospitalisation was typically even higher [26]. Similar findings were then confirmed and repeated in 5–11-year-olds [27–29], with the difference that the reports on VE against Omicron were smaller than against Delta [27–32]. Analysis of the waning of the protective effect showed a relatively rapid decline in effectiveness against symptomatic disease, within several weeks of vaccination [33]. VE against severe forms of the COVID-19 that require hospitalisation and death lasts longer, but also wanes gradually [33].

In the UK, policy decisions on vaccinating CYP were driven by scientific evidence on the health risks and benefits of the vaccine in CYP, which took time to obtain, as well as considerations of their potential positive impact on school attendance and overall well-being of children [34–36].

The Vaccine Adverse Event Reporting System (VAERS) is the national vaccine safety monitoring system in the USA accepts reports of adverse events after vaccination [37]. A study of VAERS reports relating to BNT162b2 vaccination in 12–17-year-olds in the USA from 14 December 2020 to 16 July 2021 found that the most commonly reported conditions and diagnostic findings among reports of serious events were consistent with a diagnosis of myocarditis (chest pain (56.4%), increased troponin levels (41.7%), myocarditis (40.3%), increased C-reactive protein (30.6%), and negative SARS-CoV-2 test results (29.4%)) [37]. We recently contributed to the methodological aspects of these types of studies by clarifying and advancing them [38,39].

In Scotland, the general population of 12–17-year-olds was initially offered only a single dose of BNT162b2 mRNA vaccine, but the Joint Committee on Vaccination and Immunisation recommended a second dose in November 2021 [40–43]. COVID-19 vaccination was extended to all 5–11-year-olds starting 19 March 2022 [42,43] (Table S1 in the **Online Supplementary Document**), with only a very small number of immune-suppressed children having been vaccinated earlier. Our previous study among 12–17-year-olds was the first national-level study in this age group that assessed vaccine uptake, safety, VE, and waning [8]. It allowed us to introduce some methodological improvements to reporting of vaccine uptake at the national level [9], selecting the most informative positive and negative controls for self-controlled case series (SCCS) exploring vaccine safety [38], and understanding and reporting odds ratios as rate-ratio estimates in case-control analyses [39].

Few large-scale population studies have examined both safety and VE specifically for 5–11-year-olds during the Omicron-dominant period in a setting with low vaccine uptake. Here we extended our analyses to the age group 5–11 years in Scotland. We designed a protocol to assess the ‘real-world’ effectiveness of the COVID-19 vaccination programme [44]. Our objectives concerning the vaccination of children aged 5–11 years were to: investigate uptake rates for the first and second doses; evaluate the safety profile of the vaccine after each dose; measure VE against symptomatic disease caused by the Omicron variant; and investigate waning of VE over time. Our outcome of interest was symptomatic disease based on reverse transcription-polymerase chain reaction (RT-PCR) positivity among children with respiratory symptoms, whereby asymptomatic infections were not included.

## METHODS

Details on this national prospective cohort study, including the cohorts included in each analysis, the primary data sources, data flow diagrams, and key dates corresponding to the timeframes of various analyses, are provided in Figures S1 and S2, and Table S1 and S2 in the **Online Supplementary Document** [45–48]. In brief, we studied three different cohorts of children to answer three different research questions. The first cohort was used to study vaccine uptake and it included all children aged 5–11 years in Scotland; the second was used for the safety analysis and included vaccinated children with adverse events of special interest (AESI); and the third was used to examine VE and immunity waning, and it consisted of children with symptomatic COVID-19 diagnosed according to International Classification of Disease, 10th edition (ICD-10) codes and confirmed by RT-PCR.

### Vaccine uptake

The analysis of vaccine uptake utilised data from the Turas Vaccination Management Tool, with information current as of 16 April 2024 (**Table 1**). The denominator (n = 392 658) represented the number of children aged 5–11 years in March 2022 that were included in the Early Pandemic Evaluation and Enhanced Surveillance of COVID-19 (EAVE II) cohort at its inception [45]. The vaccine uptake figures reported here reflect a cross-sectional assessment of children aged 5–11 linked to EAVE II as of March 2020. These figures may differ slightly from those published on the Public Health Scotland (PHS) website, which reports cumulative vaccine uptake based on the age of children at the time of vaccination [9].

**Table 1:** Uptake of BNT162b2 vaccine among 5–11-year-old children in Scotland at 1 January 2023

		5–11-year-olds from 19 March 2022		
**Dose**	**Sex**	**Number vaccinated**	**Population**	**Uptake**
First dose	Females	48 647	191 189	25.4%
	Males	50 577	201 469	25.1%
	Total	99 224	392 658	25.3%
Second dose	Females	31 097	191 189	16.3%
	Males	32 489	201 469	16.1%
	Total	63 586	392 658	16.2%

The uptake figures include a very small minority of children who were vaccinated prior to the study period (*i.e.* before 19 March 2022). This subset of children had specific individual or household vulnerabilities, leading to their earlier eligibility for vaccination and adherence to a different schedule for follow-up doses (Table S2 in the **Online Supplementary Document**). These vulnerabilities encompassed conditions such as chronic respiratory disease, chronic heart conditions, chronic disorders of the kidney, liver, or digestive system, chronic neurological conditions, endocrine disorders, spleen dysfunction, severe genetic abnormalities, and various forms of immunosuppression (Tables S2 and S3 in the **Online Supplementary Document**).

### Vaccine safety

The safety of the BNT162b2 vaccine in 5–11-year-olds was assessed using national data on hospital admissions and general practice consultations through a SCCS design. This analysis included all vaccinated 5–11-year-olds in Scotland and those at increased risk of severe COVID-19. We selected twenty-nine potential AESI for inclusion in this study based on recommendations from the World Health Organization [49], the Safety Platform for Emergency vACcines [50], outcomes previously monitored for influenza vaccinations [51], and input from clinicians at PHS and the University of Edinburgh. These 29 AESI were grouped into 17 health outcomes by PHS clinicians based on similarities in disease processes and outcomes (Table S4 in the **Online Supplementary Document**). The AESIs were determined from ICD-10 codes in hospitalisation records only and not on any other clinical adjudication. As a negative control outcome used to detect potential residual confounding, we evaluated the association of vaccination and SARS-CoV-2 exposure with hospital admissions for poisoning in 5–11-year-olds, which was assumed to be unrelated to vaccination or infection. [38] Each health outcome was defined using ICD-10 diagnostic codes [52]. Patients aged 5–11 years with a hospital admission containing an AESI diagnosis were identified using the Scottish Morbidity Record 01 national admission data set [53] (Tables S4 and S5 in the **Online Supplementary Document**).

We calculated the number of hospital admissions during the baseline period (75 to 15 days prior to the first dose of BNT162b2 vaccination) and within specified risk periods following vaccination for each individual and health condition, with both vaccine doses included in the same model as exposures (**Table 2**). We defined risk periods following vaccination so that they could not overlap with each other: if a child received a second dose while still in a risk period following their first dose, their follow-up was censored on the date of their second dose. We performed a SCCS analysis to investigate the temporal association between the first and second doses of BNT162b2 and 17 health outcomes in 5–11-year-olds in Scotland (Table S5 in the **Online Supplementary Document**). We calculated incidence rate ratios (IRRs) to compare the rate of hospital admissions containing a health outcome during the risk period after vaccination to the baseline period, with an IRR greater than 1 indicating a higher rate of hospitalisation following vaccination. We used the first event in each observation period following a vaccine dose. Both new cases and exacerbations of pre-existing conditions were included in the analysis. We limited the SCCS analysis to outcomes with at least five hospital admissions during the risk period containing a specific health condition to minimise risk of patient disclosure. 

**Table 2:** The number of hospital stays for each health outcome in the risk periods following the first and the second dose of BNT162b2 vaccine*

Health outcome and risk period in days†	Number of hospital stays during the baseline period	Number of hospital stays following dose 1 and 2
**Type 1 diabetes**		
Baseline: −75 to −15 d. Vaccine: 1 to 90 d.	21	28
**Vasculitis and inflammatory conditions (*e.g.* multisystem inflammatory syndrome, Kawasaki disease)**		
Baseline: −75 to −15 d. Vaccine: 0 to 42 d.	6	<5
**Seizures**		
Baseline: −75 to −15 d. Vaccine: 0 to 6 d.	29	<5
**Chronic fatigue syndrome, fibromyalgia**		
Baseline: −75 to −15 d. Vaccine: 0 to 90 d.	0	0
Baseline: −75 to −15 d. Vaccine: 90 to 180 d.	0	0
Baseline: −75 to −15 d. Vaccine: 180 to 365 d.	0	0
**Demyelination**		
Baseline: −75 to −15 d. Vaccine: 0 to 42 d.	<5	0
Baseline: −75 to −15 d. Vaccine: 42 to 90 d.	<5	0
**Thrombocytopenia**		
Baseline: −75 to −15 d. Vaccine: 0 to 21 d.	<5	0
Baseline: −75 to −15 d. Vaccine: 21 to 42 d.	<5	<5
**Arthritis**		
Baseline: −75 to −15 d. Vaccine: 0 to 42 d.	0	0
Baseline: −75 to −15 d. Vaccine: 42 to 90 d.	0	0
**Neuropathy, encephalitis, myelitis, facial palsy**		
Baseline: −75 to −15 d. Vaccine: 0 to 7 d.	<5	0
Baseline: −75 to −15 d. Vaccine: 7 to 42 d.	<5	<5
Baseline: −75 to −15 d. Vaccine: 42 to 90 d.	<5	0
**Narcolepsy**		
Baseline: −75 to −15 d. Vaccine: 0 to 42 d.	0	0
**Thrombosis and embolism**		
Baseline: −75 to −15 d. Vaccine: 0 to 21 d.	0	0
Baseline: −75 to −15 d. Vaccine: 21 to 42 d.	0	0
**Haemorrhagic stroke**		
Baseline: −75 to −15 d. Vaccine: 0 to 21 d.	0	0
Baseline: −75 to −15 d. Vaccine: 21 to 42 d.	0	0
**Autoimmune thyroiditis**		
Baseline: −75 to −15 d. Vaccine: 0 to 42 d.	0	0
Baseline: −75 to −15 d. Vaccine: 42 to 90 d.	0	0
**Myocarditis and pericarditis**		
Baseline: −75 to −15 d. Vaccine: 0 to 42 d.	0	0
**Anaphylaxis**		
Baseline: −75 to −15 d. Vaccine: 0 to 1 d.	0	0
**Guillain-Barre syndrome**		
Baseline: −75 to −15 d. Vaccine: 0 to 42 d.	0	0
Baseline: −75 to −15 d. Vaccine: 42 to 90 d.	0	0
**Myasthenia gravis**		
Baseline: −75 to −15 d. Vaccine: 0 to 42 d.	<5	0
**Disseminated intravascular coagulation**		
Baseline: −75 to −15 d. Vaccine: 0 to 21 d.	0	0
Baseline: −75 to −15 d. Vaccine: 21 to 42 d.	0	0

d – day

*Hospital stay counts n <5 have been suppressed in accordance with statistical disclosure procedures.

†Day of vaccination is day 0.

### Vaccine effectiveness and waning

To investigate VE and vaccine waning, we utilised the EAVE II platform, which integrates data on vaccinations, primary care records, and RT-PCR testing for children and adolescents aged 5–11 years in Scotland [45–48]. The primary endpoint of this analysis was symptomatic COVID-19 confirmed by a positive RT-PCR test for SARS-CoV-2. The estimates are derived from a test negative design (TND) among all CYP who were aged 5–11 at their specimen date and who were hospitalised with a respiratory condition (ICD codes beginning with ‘J’). On 1 January 2023, 99 224 children had received at least one vaccine by 1 January 2023. The median number of days between last vaccine dose and positive test for those who were vaccinated at time of their test was 302 days.

This study had only one distinct period based on the predominant SARS-CoV-2 variant circulating in the community: the Omicron period [48]. It spanned from 20 December 2021 to 18 April 2022 [48], so all positive RT-PCR tests between 15 January 2022 and 18 April 2022 were attributed to Omicron (BA.1 or BA.2), with no specific viral genotyping performed.

The analysis focussed on the BNT162b2 vaccine in a two-dose regimen. In Scotland, most children followed a two-dose schedule, except for immunocompromised individuals who were eligible for a three-dose regimen. We excluded children aged 5–11 years who received a vaccine dose before 19 March 2022. The TND estimated odds ratios comparing unvaccinated individuals with those in post-first and post-second dose vaccination periods, measured in weeks. The regression model incorporated vaccine status, sex, urban/rural classification, socioeconomic status (using quintiles of the Scottish Index of Multiple Deprivation), previous positive test history, number of QCovid risk groups [8], and a spline in days since study start. A total of 3232 children in the cohort had missing information on their residential location, from which urban/rural classification and Scottish Index of Multiple Deprivation quintile are derived. These children were excluded from the TND analysis, but they were not excluded from the SCCS analysis. We calculated VE relative to the unvaccinated, with VE defined as ‘1 − odds ratio’ from the logistic regression. Details on the statistical model and variables used to examine vaccine waning are provided in Table S2 in the **Online Supplementary Document**.

### Reporting and availability of code

We reported our findings per the STROBE and RECORD statements (Table S6 in the **Online Supplementary Document**). We provide details on the availability of data at the end of this manuscript.

### Patient and public involvement

The EAVE II Public Advisory Group reviewed the analysis design for this project. In response to their suggestions relevant to this work, we provided details on any excluded groups or aggregations and separately reported vaccine uptake in 5–11-year-olds from that among older children.

### Role of the funding source

The funders did not have any influence on the study design, analyses, interpretation, or decision to publish its findings.

## RESULTS

### Vaccine uptake

**Figure 1:**
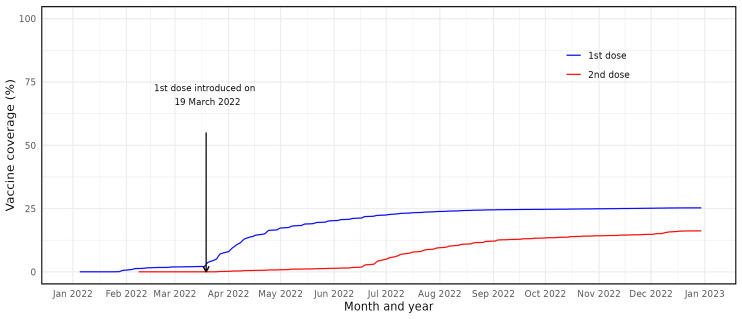
Visualisation of the uptake of BNT162b2 vaccine among 5–11-year-olds in Scotland: blue lines represent first dose uptake, red lines second dose uptake.

There were 350 300 children aged 5–11 years residing in Scotland between 6 August 2021 and 1 March 2022 (**Table 1**). By 1 January 2023, 25.1% of boys and 25.4% of girls in this age group had received their first dose, while 16.1% of boys and 16.2% of girls had received their second dose (**Figure 1**).

### Vaccine safety

There were no AESIs with increased incidence post vaccination, although the numbers were so small for most conditions that it is also possible that much larger studies would be needed to detect meaningful differences. Regardless, **Table 2** indicates that even if there was a true increase in incidence post-vaccination, it would still be small, and the AESI conditions would still be rare. Among 5–11-year-olds, 16 of the 17 health outcomes studied had fewer than five hospital admissions during the corresponding risk periods following both the first and second doses of the BNT162b2 vaccine (**Table 2**). For conditions with more than five admissions either in the pre- or post-vaccine period, we conducted an SCCS analysis. A total of 10 hospital stays for type 1 diabetes were recorded within 1–90 days of the first or the second dose (**Table 2**). However, no significant increase in hospitalisation rates (IRR = 0.66; 95% confidence interval (CI) = 0.36, 1.23) (Table S5 in the **Online Supplementary Document**). For all other conditions, the combined number of individuals contributing to these hospital stays following vaccination with either one of the doses was fewer than five. No hospital stays with myocarditis or pericarditis were recorded within 1–42 days of either the first or the second dose (**Table 2**). As a negative control, we examined hospital admissions with poisoning within 1–90 days of vaccination and found no increased risk during this period (Tables S4 and S5 in the **Online Supplementary Document**).

### Vaccine effectiveness and waning of the protective effect

During the Omicron period, the first dose in the 5–11-year-old age group resulted in a 60.8% reduction (95% CI = 0.3, 84.5) in symptomatic COVID-19 cases 2–26 weeks after vaccination. However, there was little protection from 27 weeks onwards, with VE being 13.6% (95% CI = −79.4, 58.4) (**Table 3**). Following the second dose, VE was 41.6% (95% CI = −89.6, 82.0) in the period 2–26 weeks. In the weeks ≥27, this was followed by the VE estimate of 4.1% (95% CI = −87.0, 50.8) (**Table 3**). The findings presented in **Table 3** indicate that there is a true protective effect of the vaccine, but also that the effect wanes beyond six months.

**Table 3:** Effectiveness of the BNT162b2 in 5–11-year-olds*

5–11-year-olds		Omicron period	
**Vaccine status (dose, week)**	**n**	**Events**	**VE %, 95% CI)**
None	4692	418	
1st, 2 − 26	104	5	60.8 (0.3, 84.5)
1st, ≥27	186	8	13.6 (−79.4, 58.4)
2nd, 2–26	86	3	41.6 (−89.6, 82.0)
2nd, ≥27	219	10	4.1 (−87.0, 50.8)

CI – confidence interval, VE – vaccine effectiveness

*Odds ratios were estimated using a logistic regression model. The model contains adjustments for sex, socioeconomic status, urban/rural classification, time since last positive test, number of QCovid risk groups and a spline in days since study start. ‘n’ is the number of persons contributing to each sub-sample; ‘events’ is the number of episodes of symptomatic infections with positive tests that were recorded among those persons in each sub-sample).

## DISCUSSION

We found that the nationwide vaccination programme using BNT162b2 vaccine among 5–11-year-olds in Scotland achieved low levels of coverage during the year 2022 – slightly over 25% with the first dose and just over 16% with the second dose. These rates were much lower than those achieved in 16–17-year-olds and 12–15-year-olds in 2021 [8]. While the vaccine was safe for the children in the age group of 5–11, the effectiveness against Omicron infection was 61% after the first dose and 42% after two doses. Furthermore, it was difficult to demonstrate significant positive effects beyond 26 weeks post-vaccination. Clearly, effectiveness against symptomatic Omicron infections was demonstrated even in a reasonably smaller sample where all available test results were used, but the effect did not last beyond 26 weeks.

Several factors need to be considered when interpreting these findings. The first is that the numbers available for the VE analysis in this study were smaller than they were in our previous study of 12–17-year-olds. Part of the reason is that mass testing in Scotland ended only a few weeks after 5–11-year-olds were first offered vaccinations. The campaign started from 19 March 2022, but lateral flow tests were discontinued from 18 April 2022 and RT-PCR testing sites closed from 30 April 2022. To mitigate the problem of small sample sizes, our TND analysis looked at COVID-19 RT-PCR tests among those hospitalised with a respiratory condition. This allowed us to extend the study period after the end of mass testing because individuals admitted to hospital with respiratory conditions were routinely given COVID-19 tests. To 10 September 2024, only 569 of 5–11-year-olds had positive tests, that occurred >14 days after being vaccinated, included in the TND. This allowed 14 days for the vaccination to take effect. This is why we could conduct the vaccine uptake and vaccine safety analysis reported here at the same level as we did for the older age groups in Scotland, while our VE and waning analyses are more limited in comparison [8].

A smaller number of children receiving the second *vs*. the first dose could also be explained by several factors. The initial single-dose schedule may have been influenced by perceptions of the necessity of a second dose, perceived low risk of severe illness in youngest children and during the Omicron era, increased awareness of reports regarding suboptimal VE in preventing transmission, concerns about potential side effects, and waning of vaccine-induced protection over time [46,47,54–57]. Moreover, a recent previous SARS-CoV-2 infection may have delayed vaccination and impacted vaccine uptake [57].

There are several implications of low uptake observed in this age group. Policymakers should discuss whether future efforts should primarily target high-risk children or high-risk families; moreover, whether seasonal boosting might be a better strategy for this age group; and also, how does perception of risk as low, widespread misinformation and depleted trust be addressed to increase the uptake in the future pandemics. Concerns over ‘long COVID’ may be a key rationale for vaccinating children, but they remain largely unrecognised in general population. Additionally, hybrid immunity obtained through prior infection and one dose of vaccine may have also contributed to plateauing uptake. The vaccine uptake figures reported here were based on a cross-sectional uptake among children aged 5–11 in March 2022 and links to the EAVE II cohort. For this reason, they slightly differ from the figures published on the PHS website, which reports cumulative vaccine uptake by the age of the children at the date of vaccination. Therefore, those two sets of figures are not directly comparable, although the difference is unlikely to be substantial, as we discussed elsewhere [9].

While the UK Medicines and Healthcare products Regulatory Agency has evaluated the BNT162b2 vaccine and confirmed its safety for use in 5–11-year-olds [58], it continues to be monitored as it is being administered to the wider population. While the overall evidence remains reassuring, some reports of potential side effects, including mild cases of myocarditis and myopericarditis in young individuals, have been noted (**Box 1**), albeit not in 5–11-year-old children [14,38,39,47,59]. Our analysis of general practice consultations and hospitalisations revealed no safety concerns, which is consistent with expectations, but we cannot exclude the possibility of very rare serious adverse events: while myocarditis and other AESIs were clearly rare, some could have been missed due to limited statistical power of this study to detect them. Myocarditis and pericarditis were the most frequently reported concerns in previous research among the children aged 12–17 years [13–18], but not among 5–11-year-olds, which is consistent with our findings. Vaccination is generally effective in preventing symptomatic COVID-19 within the first few months after administration. However, during the Omicron period in Scotland among 5–11-year-olds, protection against symptomatic disease was relatively short-lived.

In Scotland, hospitalisations containing the 17 health outcomes assessed in 5–11-year-olds following the first and second doses of the BNT162b2 vaccine were exceptionally rare (**Table 2**). No hospital stays for myocarditis or pericarditis were recorded in our sample after either the first or the second dose. These findings align with the results of a randomised, placebo-controlled, observer-blinded phase 3 trial that evaluated the safety of the BNT162b2 vaccine in healthy children aged 5–11 years [21]. Hospitalisations are generally infrequent in this age group, which limits the opportunities to perform SCCS analyses [60,61]. Additionally, we did not observe any general practice consultations or hospitalisations for myocarditis or pericarditis after vaccination. The health outcome category for vasculitis and inflammatory conditions includes paediatric inflammatory multisystem syndrome, a now recognised condition that occurs weeks after infection with the virus causing COVID-19 [62]. No significant increase in hospitalisations for vasculitis or inflammatory conditions was detected following vaccination.

We performed our study at the national level, so it benefited from a large sample size, allowing us to identify patterns and effects related to the uptake and safety of the vaccine. Future research will require larger samples, possibly obtained through meta-analyses of several national-level studies, to reduce the substantial uncertainty around the estimates. Previous research utilising the EAVE II platform has demonstrated the value of integrating data from multiple sources, enabling detailed and timely analyses of the pandemic which have informed policy decisions almost in real time [45–47]. However, there are specific challenges and limitations associated with studying COVID-19 in CYP. One particular subgroup of CYP in Scotland (n = 8300) was classified as especially vulnerable and at high risk from COVID-19 [8]. This group was vaccinated alongside adults and prior to most other CYP using vaccines licensed for adults at the time. Consequently, they are not directly comparable to the broader CYP population due to their higher baseline risk for COVID-19 complications, differing vaccination timelines, exposure to different SARS-CoV-2 variants, and likely fewer overall exposures due to shielding measures. Additionally, they received different vaccine formulations and dosages. For these reasons, this subgroup was excluded from the VE analyses, but was included in the safety analyses given its relevance to vaccine safety outcomes.

In the safety analysis, we included a 15-day ‘washout’ period prior to vaccination in the SCCS to avoid introducing bias, where people who have adverse events are less likely to get vaccinated. Furthermore, the use of negative controls in SCCS analyses – applied to either exposures or outcomes – has proven to be an effective strategy for identifying potential biases and validating the assumptions necessary for reliable SCCS results [38,63–67].

Unlike the previous study on 12–17-year-olds [8], this research was not affected by potential misclassification of COVID-19 cases to specific dominant variant periods which might have otherwise impacted VE estimates, as all vaccination occurred during the Omicron period. Therefore, we had no issues differentiating between variants or with the ‘depletion of susceptibles’ bias [68].

The outcome in the TND analysis was defined by a positive RT-PCR test among individuals hospitalised with a respiratory infection. Our study lacks data on SARS-CoV-2 antibody levels, which could have been useful for studying their association with VE and waning. While it is impractical to collect this type of information in a large-scale national study, future research on smaller cohorts should investigate the associations between antibody levels, VE, waning, and clinical symptoms. It is worth noting that higher VE after dose 1 than dose 2 is an unexpected and counter-intuitive finding, but it is plausibly a result of a chance effect in a smaller sample of those who received both doses. It is also possible that children who received the second dose were less healthy than those who received the first dose.

We initially planned to examine VE against severe COVID-19 outcomes [69]. However, there was concern that hospitalisations among the children of this age during the study period might primarily reflect cases ‘with’ COVID-19 rather than ‘because of’ COVID-19, which would have potentially introduced misclassification bias. While PHS’ weekly report indicated that 60% of hospital admissions in Scotland in December 2021 were ‘because of’ COVID-19 rather than incidental cases ‘with’ COVID-19 [69], this figure applies to all age groups and may not accurately reflect the situation for the children aged 5–11 years, especially during 2022 and the Omicron period [70,71].

This analysis answers several key research questions concerning children aged 5–11 years. We utilised various data sets and employed different study designs tailored to each research question. The EAVE II collaboration has previously published a series of individual studies focussing on vaccine uptake [72], safety [73], effectiveness (VE) [46,74–77], and waning [54] among adults in Scotland, and among the children aged 12–17 years {8]. Those papers and our further methodological contributions [9,38,39] provide detailed discussions on specific aspects of investigating such research questions, including considerations of study design, biases, confounding factors, and chance effects, all of which are also relevant to this study.

## CONCLUSION

This study has several important implications for policy, practice, and future research. Vaccine uptake rates among children aged 5–11 years have been quite low and the rollout began significantly later compared to adults. The findings demonstrate that no concerning safety signals were detected for the BNT162b2 vaccine, although the study was underpowered to detect very rare events. The vaccine is also effective in reducing symptomatic infections in children. This protection, however, likely diminishes quite rapidly over time. This creates challenges for decision-making among authorities, parents, and children. Recent studies have corroborated our findings, highlighting limited effectiveness and relatively rapid waning of the two-dose BNT162b2 COVID-19 vaccine in 5–11-year-olds [78,79]. However, these studies also showed substantial VE with the first booster dose, suggesting that booster doses could be a viable strategy to enhance school attendance and minimise educational disruptions [78,79]. Booster effects are inferred from other studies, while a notable limitation of our study is the lack of data on the impact of booster doses in this age group.

There is a strong incentive to ensure children can safely return to school and maintain consistent attendance [80,81]. Vaccination could contribute to this goal to some extent, and several studies have highlighted the effectiveness of health education campaigns in developing and implementing strategies to address vaccine hesitancy among parents and adolescents [2,82–85]. However, we did not assess whether vaccination impacted school attendance or whether the severity of ‘symptomatic’ illness alone would have caused absences. In the context of current testing and isolation guidelines, many cases might go undetected, allowing children to continue attending school despite mild symptoms. Moreover, given the evidence of waning immunity observed in this study, any potential effect of vaccination on improving school attendance might be limited to only several months, creating challenges for policymakers. Nevertheless, regarding pandemic preparedness and intervention scenarios, a safe and effective vaccine could keep primary school children safe at school while the rest of the population are vaccinated. Reducing educational disruption is just one consideration in the decision to vaccinate children of this age. A key factor is the potential impact on transmission, which remains insufficiently understood. Further research is needed to explore whether vaccines provide more durable protection against severe disease compared to symptomatic infection. This understanding will be crucial in shaping future policies.

## Additional material


Online Supplementary Document
