# Supplementary material for: BNT162b2 COVID-19 vaccination uptake, safety, effectiveness, and waning in children and young people aged 5–11 years in Scotland
Source: J Glob Health. 2025 Aug 29;15:04250. doi: 10.7189/jogh.15.04250 (PMC12395862; doi:10.7189/jogh.15.04250)
Supplement: Online Supplementary Document [file jogh-15-04250-s001.pdf]

Supplement to: Rudan I, Kerr S, Sullivan C, Jeffrey K, Grange Z, Fenton L, Kurdi A, Shi T, Cullen L, Simpsom CR, Katikireddi SV, Ritchie SLD, Robertson C, Sheikh SA. BNT162b2 COVID-19 vaccination uptake, safety, effectiveness, and waning in children and young people aged 5–11 years in Scotland. J Glob Health. 2025;15:04250.

(i) **Table S1.** Timeline of key events in Scotland related to vaccination of children and young people (CYP).

(ii) **Figure S1.** Data diagram of primary data sources.

(iii) **Figure S2.** The number of children and young people included in the study of the vaccine uptake.

(iv) **Table S2.** Further information on primary data sources.

(v) **Table S3.** Clinical and other risk groups for children and young people aged 12-17 years.

(vi) **Table S4.** List of conditions that were investigated to establish vaccine safety and their corresponding International Classification of Diseases (ICD)-10 codes and defined risk periods (in days).

(vii) **Table S5.** A detailed description of datasets, procedures and methods used to explore BNT162b2 vaccine safety in children and young people in Scotland.

(viii) **Table S6.** The STROBE and RECORD statements.

(ix) **References** to supplementary online material.

Table S1. Timeline of key events in Scotland related to vaccination of children and young people (CYP).

Date	Technical notes issued by the UK or Scottish Government	Recommendations
09/07/2021 - The Conditional Marketing Authorisation for Pfizer-BioNTech BNT162b2 came into effect on 9 July 2021, with approval previously being provided under Regulation 174. Joint Committee on Vaccination and Immunisation (JCVI) advises that only UK-authorised COVID-19 vaccines should be offered to those aged less than 18 years. At this time, the Pfizer-BioNTech BNT162b2 vaccine is the only vaccine authorised for persons aged 12 to 17 years in the UK.		
06/08/2021	1.https://www.gov.scot/news/vaccinations-for-16-to-17-year-olds/<br><br>2.https://www.gov.uk/government/publications/jcvi-statement-august-2021-covid-19-vaccination-of-children-and-young-people-aged-12-to-17-years/jcvi-statement-on-covid-19-vaccination-of-children-and-young-people-aged-12-to-17-years-4-august-2021<br><br>3.https://www.nhsinform.scot/covid-19-vaccine/the-vaccines/vaccinating-children-and-young-people-aged-12-to-17-years/<br><br>4.https://www.gov.uk/government/publications/regulatory-approval-of-pfizer-biontech-vaccine-for-covid-19/summary-of-product-characteristics-for-covid-19-vaccine-pfizerbiontech	**VACCINATION FOR 16 TO 17-YEAR-OLDS**<br><br>All young people 16 to 17 years of age will now be offered the coronavirus (COVID-19) vaccination in Scotland. In line with the latest advice from the Joint Committee on Vaccination and Immunisation (JCVI), they will be offered a first dose of the Pfizer-BioNTech vaccine. From **Friday 6 August**, people who are 16 or 17 in mainland Scotland will be invited to register their interest through the online portal at NHS Inform, and will then be sent an appointment via SMS or email. Eligible young people in Shetland, Orkney and Western Isles will be contacted by their health board and invited to attend clinics.<br><br>Alternatively, drop-in clinics will be available for 16 to 17-years-olds. The start date for clinics opening for this age group will be confirmed shortly. Anyone who doesn't register an interest or attend a drop-in clinic, once open, will be sent an appointment invitation through the post. It is expected that everyone in this age group will have been offered a vaccination appointment by the end of September.<br><br>**ADDITIONAL AT-RISK GROUPS OF MINORS**<br><br>In addition to 16 to 17-year-olds, we have started offering COVID-19 vaccinations to children and young people who are from key groups; and are between 12 to 17 years. Vaccinations will be offered to:<br><br>12 to 15-year-olds with severe neuro-disabilities<br><br>12 to 15-year-olds with Down's syndrome<br><br>12 to 15-year-olds with underlying conditions resulting in immunosuppression<br><br>12 to 15-year-olds with profound and multiple learning disabilities (PMLD)<br><br>12 to 15-year-olds with severe learning disabilities<br><br>12 to 17-year-olds who have a diagnosed learning/intellectual disability (mild or moderate)

		16-year-olds who have any of these above conditions or underlying conditions that place them at higher risk of serious COVID-19 (that were not 16 at the time of the previous invite for all 16 and 17-year-olds in March 2021)<br><br>young people aged 12 years and above who live in the same household of persons (adults or children) who are immunosuppressed (we previously invited over 16s who are household contacts of those on the shielding list)
20/09/2021	1.https://www.gov.scot/news/vaccinations-for-12-15-year-olds/<br><br>2.https://www.gov.scot/publications/coronavirus-covid-19-update-first-ministers-statement-14-september-2021/<br><br>3.https://www.nhsinform.scot/covid-19-vaccine/the-vaccines/vaccinating-children-and-young-people-aged-12-to-17-years/	**VACCINATION FOR 12 TO 15-YEAR-OLDS**<br><br>Children and young people aged 12 -15 years old will be offered a dose of the coronavirus (COVID-19) vaccination from **Monday 20 September** after Scottish Ministers accepted advice from the four UK Chief Medical Officers (CMOs). As a result, a dose of Pfizer-BioNTech vaccine will be offered to all children and young people aged 12-15 who are not already covered by existing advice from the Joint Committee on Vaccination and Immunisation (JCVI) in a move to reduce the disruption caused to education by COVID-19. This group will be offered their injections in drop-in clinics and community settings followed by each young person receiving a letter inviting them to attend a community clinic. For some rural Health Boards, those aged 12 to 15 will first be offered the vaccine at school.<br><br>Following the initial phase, vaccines will be offered in both communities and schools so that anyone who hasn't been vaccinated, but would like to be, has opportunity to take up the offer. Meanwhile, people who received their vaccination during phase one of the national COVID-19 vaccination programme in Scotland will start to receive booster injections **from 20 September**. This follows advice from the JCVI which has advised that the booster dose can be given alongside the flu jab and should be offered no earlier than six months after completion of the primary vaccine course. Frontline health and social care workers will be able to book their appointment online at NHS Inform from 20 September and from that date, residents in care homes for older people will be offered both flu and COVID-19 booster vaccination.<br><br>Children aged 12-15 who have specific underlying conditions or disabilities are already covered by previous JCVI advice and will be offered two doses, eight weeks apart.<br><br>**DETAILS OF PROCEDURES**<br><br>NHS Scotland is offering 2 doses of the coronavirus vaccine to all children and young people aged 12 to 17 years. All children and young people aged 16 and 17 years are eligible for a booster dose. Some children and young people at higher risk from coronavirus are also eligible for additional doses (third primary dose and/or booster doses).<br><br>Children and young people aged 12 to 17 years, who have recently tested positive for coronavirus, should wait 12 weeks after the date they were tested to get the vaccine (any dose). However,

		those aged 12 to 17 and at higher risk from coronavirus, can have your coronavirus vaccine from 4 weeks after coronavirus infection.<br><br>Children and young people aged 12 to 17 years were offered a second dose of the vaccine from 12 weeks after the first dose. All young people aged 16 or 17 years were eligible for a booster dose. Some children and young people aged 12 to 15 years at higher risk from coronavirus were eligible for a booster dose, from 12 weeks after their second dose. This includes those: (i) who are at increased risk from coronavirus due to underlying health conditions; (ii) who share living accommodation, on most days, with someone who has a weakened immune system; (iii) with a severely weakened immune system who have had a third primary dose.<br><br>Children and young people aged 12 to 17 years with a weakened immune system were also offered a spring booster dose of the coronavirus vaccine. The spring booster dose was usually offered around 6 months (and not before 3 months) since the last dose of the coronavirus vaccine. Children and young people aged 12 to 17 years who were at increased risk from coronavirus were offered 2 doses of the vaccine, given 8 weeks apart. This included those who: (i) were at increased risk from coronavirus due to underlying health conditions; (ii) shared living accommodation, on most days, with someone who has a weakened immune system; (iii) were aged 16 or 17 years who were an unpaid carer or a frontline health or social care worker.<br><br>Children and young people aged 12 to 17 years with a severely weakened immune system were offered 3 primary doses of the coronavirus vaccine. They were also eligible for a booster dose to help improve protection.
22/02/2022	1. Available from: https://www.gov.scot/news/vaccinations-for-all-five-to-11-year-olds/<br><br>2.https://www.nhsinform.scot/covid-19-vaccine/the-vaccines/vaccinating-children-aged-5-to-11-years/<br><br>3.https://www.gov.uk/government/news/mhra-approves-the-moderna-covid-19-vaccine-spikevax-for-use-in-6-to-11-year-olds	Children aged 5 to 11 will be offered COVID-19 vaccination appointments in community clinics from 19 March 2022. This follows the advice last week from the Joint Committee on Vaccination and Immunisation (JCVI) recommending the universal vaccination of 5-11 year-olds. Appointments will be scheduled according to age with 11 years olds invited first, followed by those aged eight to 10 and then those between five and seven years old. Where there are siblings efforts will be made to invite them to back-to-back appointments to avoid multiple trips for families. Details of how to rearrange unsuitable appointments will be included in the letters.<br><br>Children in this age group who have specific medical conditions which place them at greater risk from COVID-19 are already being vaccinated and will continue to be prioritised. Vaccinations for all 5-11 year-olds will be delivered alongside second booster jabs for those aged 75 and over and those in specific at risk cohorts following yesterday’s recommendation from the JCVI. These individuals will be invited as they become eligible from 24 weeks after their last booster with the first groups receiving blue envelopes with appointments from the second week in March.

Figure S1. Data diagram of primary data sources.

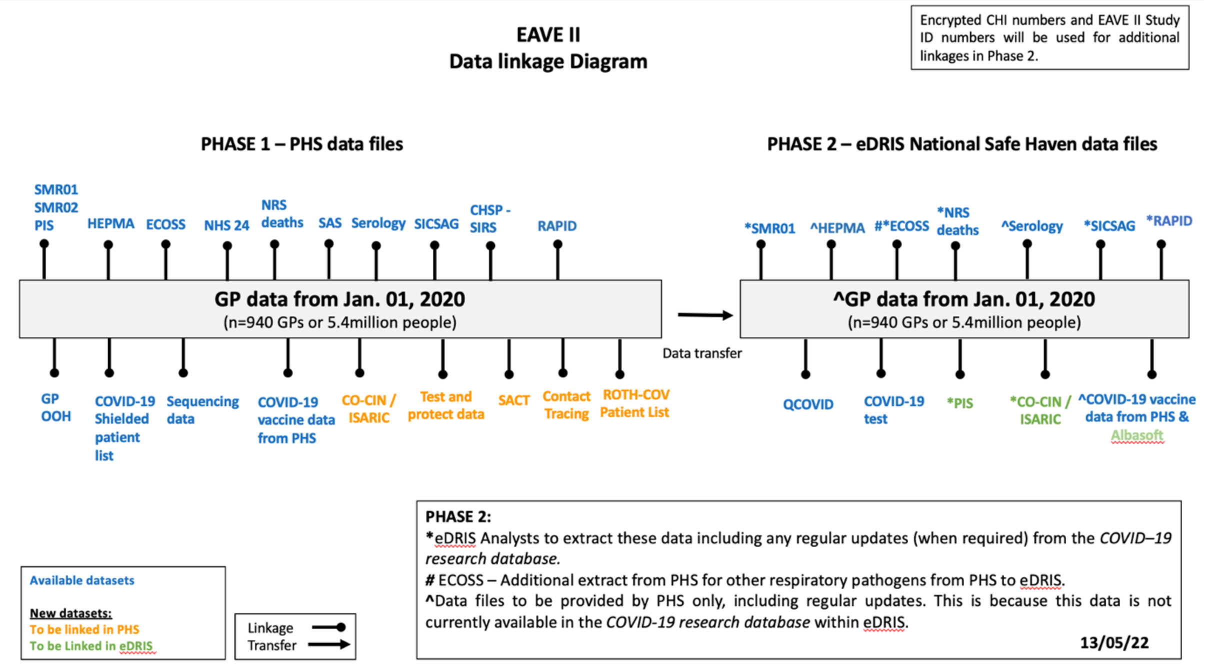

Figure S2: The number of children aged 5-11 years included in the study of the vaccine uptake. The number of 350,300 in the main text represents an estimate of the number of children aged 5-11 years in Scotland, because the number of records in the EAVE II cohort is larger than the population of Scotland as leavers were unaccounted for. People who have interacted with the health service (e.g. had a hospital admission, or a vaccination) are given larger weight, while those with no known interactions with the health service are down-weighted to derive this estimate.

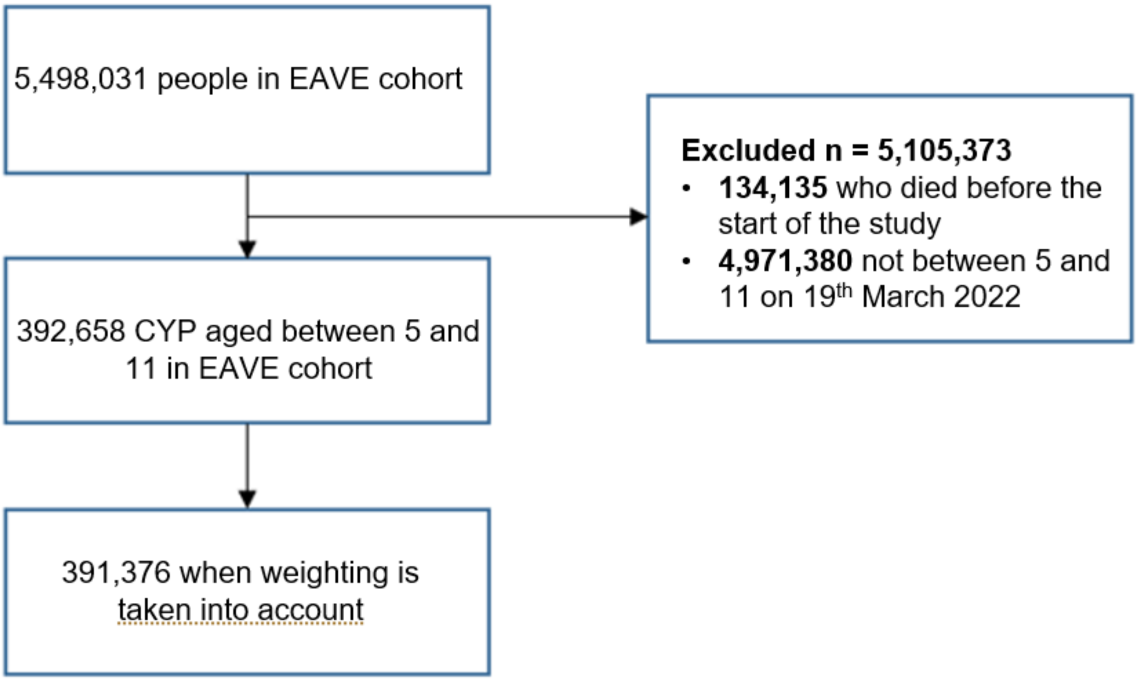

Table S2. Further information on primary data sources.

Primary data sources for the EAVE II database	The residents in Scotland, including CYP, are universally registered with primary care, which provides a comprehensive array of healthcare services free at the point of care. Access to secondary care is typically through a general practitioner (GP) based within a primary care practice or via the emergency department (ED) or out-of-hours primary care services. During the acute phase of the pandemic, community-based COVID-19 hubs (a GP-led service designed to segment patients and reduce the risk of nosocomial infections) were established. We used anonymised individual patient-level data from general practices, hospitals, death registries, and RT-PCR testing (including NHS and UK Government testing centre data – Pillar 1 and 2, respectively) to investigate the changing dynamics of COVID-19 in Scotland.<br><br>We used data from all 940 Scottish primary care practices. Clinical data collected by primary care in Scotland have consistently been shown to be of high quality (90% completeness and accuracy) and their value for epidemiological research has been repeatedly demonstrated [45-48]. These were linked to other datasets including the Electronic Communication of Surveillance in Scotland ECOSS (all virology testing national database), the Scottish Morbidity Record (hospitalisation including intensive care unit (ICU) stay), and National Records Scotland (death certification).
Summary of key dates and events in relation to the conducted analyses	Vaccine effectiveness data ranges from Mar 19th 2022 to Sep 10th 2024.<br>However, almost all vaccine doses administered to children aged 5-11 years occurred before 1st Jan 2023.<br>Vaccine uptake data is taken as a snapshot on 1st Jan 2023.<br>The date of the earliest vaccination given to any minor in Scotland was Jan 25th, 2021. The "Delta period" ranges from Aug 6th, 2021, to Dec 19th, 2021. The "Omicron period" ranges from Dec 20th, 2021, to the present date.<br>Any test taken before the 1st of November 2021 is considered Delta. Any test taken from the 15th January to the present date is considered Omicron.<br>Preparations for vaccine safety monitoring at the hospitals started as early as Sep 1st, 2020.<br>Hospital admissions end date: Sep 10th, 2024<br>General practitioners' consultations end date: March 28th, 2023
Definitions for vaccine effectiveness analysis	"Day 0" for 5-11 year-olds was Mar 19th 2022, when the BNT162b2 vaccine was offered by the Scottish government.
Characteristics of CYP vaccinated before the period of the study	A total of 8,300 CYP aged 5-11 years were vaccinated before the offer of the licensed vaccine (i.e., before Mar 19th, 2022) because of particular vulnerability and a need for protection.<br>Among these children and young people, 57.0% were male and 43.0% female. About 10% do not have the type of vaccine recorded, but among those 90% who do, 100.0% chose the vaccine produced by Pfizer-BioNTech, 0.0% the vaccine by Astra Zeneca-Oxford, and 0.0% by Moderna. A total of 8,300 (100%) received the first dose, and 6,939 (86.7%) the second dose.

	These CYP are included in the uptake and safety analysis but are not included in the vaccine effectiveness analysis (VE) because of lack of comparability that was explained in the discussion of the main text of the manuscript.
Details on safety analysis	The 17 health outcomes included were: seizures, demyelination, anaphylaxis, myocarditis and pericarditis, Guillain-Barre syndrome, thrombocytopenia, neuropathy, encephalitis and myelitis, vasculitis and inflammatory conditions, chronic fatigue, narcolepsy, thrombosis and embolism, haemorrhagic stroke, autoimmune thyroiditis, myasthenia gravis, disseminated intravascular coagulation, arthritis, and type 1 diabetes.<br>Risk periods following vaccination were attributed to health outcomes by clinicians within PHS, defined as the time in which an outcome could realistically present if associated with vaccination (**Supplementary Table S4**).<br>The detailed procedures of their identification, their association with the information on vaccination and death records, treatment of unvaccinated subjects and the adjustments made when the risk period for some health outcomes was beyond the study period are described in **Supplementary Table S5.**<br>In addition to hospital admissions, we also reviewed all GP consultations for a possible diagnosis of myocarditis or pericarditis in 5-11 year-olds in Scotland following vaccination, because these diagnoses were associated with the greatest concern in the literature to date. Details of linking the information on vaccination status with GP records and the design of the SCCS analysis for myocarditis or pericarditis are presented in **Supplementary Table S5.**
Details on ECOSS data	The ECOSS data included all RT-PCR test results from both Pillar 1 (NHS labs) and Pillar 2 (the Lighthouse government lab in Glasgow). NHS labs provide information on in-hospital testing, while the Lighthouse lab provides information on community-based testing. In the TND study, only RT-PCR COVID-19 tests in individuals who were hospitalised with a respiratory infection were used.<br>The linkage to the national vaccination record within GP practices was achieved using the community health index (EAVE Study link number). The EAVE-II study was used for the QCovid clinical risk group information from GP records. [**S1,S2**] The study period was Mar 19th 2022 until Sep 10th, 2024.
Details on statistical modelling to include the full set of variables in vaccine effectiveness and waning analysis	Analysis was performed by logistic regression. The logistic regression model included vaccine status, sex, urban/rural classification, socioeconomic status measured by quintiles of the Scottish Index of Multiple Deprivation (SIMD), time since last positive PCR test, number of QCovid risk groups and a spline in days since the study start. Vaccine effectiveness was defined as “1 – odds ratio” from the logistic regression.

Table S3. Clinical and other risk groups for children and young people aged 5-11 years [**S3,S4**]

Chronic respiratory disease	Including those with poorly controlled asthma that requires continuous or repeated use of systemic steroids or with previous exacerbations requiring hospital admission, cystic fibrosis, ciliary dyskinesias and bronchopulmonary dysplasia. (poorly controlled asthma is defined as: ≥2 courses of oral corticosteroids in the preceding 24 months OR on maintenance oral corticosteroids OR ≥1 hospital admission for asthma in the preceding 24 months)
Chronic heart conditions	Haemodynamically significant congenital and acquired heart disease, or less severe heart disease with other co-morbidity. This includes:<br>• single ventricle patients or those palliated with a Fontan (Total Cavopulmonary Connection) circulation<br>• those with chronic cyanosis (oxygen saturations <85% persistently)<br>• patients with cardiomyopathy requiring medication<br>• patients with congenital heart disease on medication to improve heart function<br>• patients with pulmonary hypertension (high blood pressure in the lungs) requiring medication
Chronic conditions of the kidney, liver or digestive system	Including those associated with congenital malformations of the organs, metabolic disorders and neoplasms, and conditions such as severe gastroesophageal reflux that may predispose to respiratory infection
Chronic neurological disease	This includes those with<br>• neuro-disability and/or neuromuscular disease that may occur as a result of conditions such as cerebral palsy, autism, epilepsy and muscular dystrophy<br>• hereditary and degenerative disease of the nervous system or muscles, other conditions associated with hypoventilation<br>• severe or profound and multiple learning disabilities (PMLD), Down's syndrome, those on the learning disability register<br>• neoplasm of the brain
Endocrine disorders	Including diabetes mellitus, Addison's and hypopituitary syndrome
Immunosuppression	Immunosuppression due to disease or treatment, including:<br>• those undergoing chemotherapy or radiotherapy, solid organ transplant recipients, bone marrow or stem cell transplant recipients<br>• genetic disorders affecting the immune system (e.g. deficiencies of IRAK-4 or NEMO, complement disorder, SCID)<br>• those with haematological malignancy, including leukaemia and lymphoma<br>• those receiving immunosuppressive or immunomodulating biological therapy<br>• those treated with or likely to be treated with high or moderate dose corticosteroids<br>• those receiving any dose of non-biological oral immune modulating drugs e.g. methotrexate, azathioprine, 6-mercaptopurine or mycophenolate<br>• those with auto-immune diseases who may require long term immunosuppressive treatments<br>Children who are about to receive planned immunosuppressive therapy should be considered for vaccination prior to commencing therapy.
Asplenia or dysfunction of the spleen	Including hereditary spherocytosis, homozygous sickle cell disease and thalassemia major
Serious genetic abnormalities that affect a number of systems	Including mitochondrial disease and chromosomal abnormalities

Table S4. List of conditions that were investigated to establish vaccine safety and their corresponding ICD-10 codes and defined risk periods (in days).

Health Outcome	Risk periods applied to outcome (days)*	Included diagnoses	ICD-10 codes of included diagnoses
Guillain-Barre syndrome	1 – 42<br>1 – 90	Disorders of other cranial nerves	G52.7
		Inflammatory polyneuropathy	G61.0
Neuropathy, encephalitis and myelitis	1 – 7<br>1 – 42<br>1 – 90	Encephalitis, myelitis and encephalomyelitis	G04.0, G04.8, G04.9
		Other acute disseminated demyelination	G36.0, G36.1
		Other demyelinating diseases of central nervous system	G37.3
		Facial nerve disorders	G51.0
		Hereditary and idiopathic neuropathy	G60.9
		Inflammatory polyneuropathy	G61.1, G61.8, G61.9
		Other polyneuropathies	G62.0, G62.2, G62.8, G62.9
		Other disorders of peripheral nervous system	G64
		Disorders of autonomic nervous system	G90.0, G90.9
		Optic neuritis	H46
		Injury of cranial nerves	S04.5
Demyelination	1 – 42<br>1 – 90	Multiple sclerosis	G35
		Other acute disseminated demyelination	G36.9
		Other demyelinating diseases of central nervous system	G37.8, G37.9
Narcolepsy	1 – 42	Sleep disorders	G47.4
Myasthenia gravis	1 – 42	Myasthenia gravis and other myoneural disorders	G70.0, G70.9
		Disorders of myoneural junction and muscle in diseases classified elsewhere	G73.3
Thrombocytopenia	1 – 21<br>1 – 42	Purpura and other haemorrhagic conditions	D69.3, D69.4, D69.5, D69.6
		Other necrotizing vasculopathies	M31.1
Thrombosis and embolism	1 – 21<br>1 – 42	Intracranial and intraspinal phlebitis and thrombophlebitis	G08
		Retinal vascular occlusions	H34.0, H34.1, H34.2, H34.9
		Pulmonary embolism	I26.0, I26.9
		Cerebral infarction	I63.6
		Other cerebrovascular diseases	I67.6

		Arterial embolism and thrombosis	I74.0, I74.1, I74.2, I74.3, I74.4, I74.5,<br>I74.8, I74.9
		Phlebitis and thrombophlebitis	I80.1, I80.2, I80.3, I80.8, I80.9
		Portal vein thrombosis	I81
		Other venous embolism and thrombosis	I82.0, I82.1, I82.2, I82.3, I82.8, I82.9
		Other disorders of kidney and ureter, not elsewhere classified	N28.0
		Complications following abortion and ectopic and molar pregnancy	O08.2
		Venous complications and haemorrhoids in pregnancy	O22.3, O22.5, O22.8, O22.9
		Venous complications and haemorrhoids in the puerperium	O87.1, O87.3, O87.8, O87.9
		Obstetric embolism	O88.2
Disseminated intravascular coagulation	1 – 21<br>1 – 42	Disseminated intravascular coagulation [defibrination syndrome]	D65
Arthritis	1 – 42<br>1 – 90	Seropositive rheumatoid arthritis	M05.0, M05.1, M05.2, M05.3, M05.8, M05.9
		Other rheumatoid arthritis	M06.0, M06.2, M06.3, M06.4, M06.8, M06.9
Autoimmune thyroiditis	1 – 42<br>1 – 90	Thyroiditis	E06.3, E06.9
		Complications of the puerperium, not elsewhere classified	O90.5
Haemorrhagic stroke	1 – 21<br>1 – 42	Subarachnoid haemorrhage	I60.0, I60.1, I60.2, I60.3, I60.4, I60.5<br>I60.6, I60.7, I60.8, I60.9
		Intracerebral haemorrhage	I61.0, I61.1, I61.2, I61.3, I61.4, I61.5<br>I61.6, I61.8, I61.9
		Other nontraumatic intracranial haemorrhage	I62.0, I62.1, I62.9
Myocarditis and pericarditis	1 – 42	Other acute ischaemic heart diseases	I24.1
		Acute pericarditis	I30.0, I30.8, I30.9
		Acute myocarditis	I40.1, I40.8, I40.9
		Cardiomyopathy	I42.7,
		Complications and ill-defined descriptions of heart disease	I51.4
		Complications of the puerperium, not elsewhere classified	O90.3
Seizures	0 – 6	Epilepsy	G40.0, G40.1, G40.2, G40.3, G40.4, G40.5,<br>G40.6, G40.7, G40.8, G40.9
		Status epilepticus	G41.0, G41.1, G41.2, G41.8, G41.9

		Convulsions, not elsewhere classified	R56.0, R56.8
Chronic fatigue	1 – 90	Other disorders of brain	G93.3
		Myositis	M60.1, M60.9
		Other soft tissue disorders, not elsewhere classified	M79.1, M79.7
Vasculitis and inflammatory conditions	1 – 7<br>1 – 21<br>1 – 72	Purpura and other haemorrhagic conditions	D69.0
		Vasculitis limited to skin, not elsewhere classified	L95.8, L95.9
		Polyarteritis nodosa and related conditions	M30.3
		Other necrotizing vasculopathies	M31.0
		Other systemic involvement of connective tissue	M35.8
		Systemic Inflammatory Response Syndrome [SIRS]	R65.2, R65.3
		Emergency use of U07	U07.5
Anaphylaxis	0 – 1	Other complications of surgical and medical care, not elsewhere classified	T88.6
Type 1 diabetes	1 – 90	Type 1 diabetes mellitus	E10.0, E10.1, E10.2, E10.3, E10.4, E10.5, E10.6, E10.7, E10.8, E10.9
**Negative control (**Poisoning)	1 – 90	Poisoning by drugs, medicaments and biological substances	T36 - T50

* Between one and three risk periods were applied for each health outcome, and for each risk period all diagnoses related to that health outcome were included.

Table S5. A detailed description of datasets, procedures and methods used to explore BNT162b2 vaccine safety in children and young people in Scotland and the results obtained.

STEP	PROCEDURE
1. Identification of patients aged 5-11 years admitted to the hospital with an AESI diagnosis using the Scottish Morbidity Record 01 (SMR01) national admission dataset	The SMR01 dataset includes data on discharge diagnoses for all inpatient and day patient episodes from acute specialties from hospitals in Scotland, excluding obstetric and psychiatric specialties. Episode level hospital data were extracted from SMR01 for the period Mar 1, 2020 to Sep 10, 2024. An episode is a period of hospital care initiated by a referral (including re-referral) or admission and ended by a discharge [**S5**].<br>AESI events were identified based on the presence of a relevant ICD-10 code within any episode and in any main or other condition diagnosis. Multiple diagnoses within a stay were only counted once for each AESI.
2. Identification of vaccinations delivered to 5-11 year-olds and linkage with AESI related hospital stays	Data on 1st and 2nd dose COVID-19 vaccine (BNT162b2) vaccinations administered to 5-11 year-olds in Scotland were extracted from the Turas Vaccination Management tool (VMT), covering the period Dec 8th 2020 to16th Apr 2024. The VMT is a web-based tool for healthcare staff in Scotland to record real-time patient vaccination data at the point of care [**S6**]. Whilst some at risk 5-11 year-olds were eligible for vaccination early in the programme, the mass roll out to 5 to 11 year olds began on Mar 19th, 2022 and this age group has predominantly received the BNT162b2 vaccine.<br><br>From Mar 19, 2022 to Jan 1, 2023, 99,224 of 5-11 year-olds received a first dose vaccine in Scotland, and 63,586 went on to have a second dose vaccine in this time period. AESI related hospital stays with admission date during the period Mar 1, 2020 to Sep 10, 2024 (described above) were linked to Scottish COVID-19 vaccination records using the unique Community Health Index (CHI) number.
3. Association of AESI diagnosis patients with death records	The linked AESI hospital stays with admission date during the period Mar 1, 2020 to Sep 10, 2024 and Scottish COVID-19 vaccination records (described above) were further linked to National Records of Scotland (NRS) death records using the unique Community Health Index (CHI) number. Data on deaths covered the period Dec 8, 2020 to Oct 4, 2024.
4. Methods for self-controlled case series (SCCS) analysis	Conditional Poisson regression models were fit, considering hospital stays stratified by individual, with an offset for the length of the risk period. Incidence rate ratios (IRRs) were estimated to quantify the rate of hospital stays for a first health outcome in the risk period following vaccination relative to the baseline period (75- to-15-days before first dose BNT162b2 vaccination). An IRR > 1 suggests an increased risk following vaccination. Individuals were censored on the earliest of the following: date of death, or (with respect to their first dose) date of second dose BNT162b2 vaccine. There was no need to censor for an end of study date, because the risk period following the last date of vaccination for any 5-11 year-olds is well before our hospitalisation data ends.
5. Treatment of unvaccinated subjects	We considered including unvaccinated 5-11 year-olds who experienced the health outcome with admission date during the period Sep 1, 2020 to Apr 30, 2022 in the SCCS analysis with an additional temporal stratification of calendar time to allow for any potential trends in the health outcomes over time. We concluded that, if they had an event before they were vaccinated, they can only possibly have follow up time in the baseline period, thus making no contribution

<table>
<tr><td></td><td colspan="4">to the likelihood function, so the estimates are identical whether they are included or not.</td></tr>
<tr><td>6. Linking vaccination records with GP records for myocarditis or pericarditis and the design of the SCCS analysis</td><td colspan="4">Patients aged 5 to 11 years old with recorded GP consultations for myocarditis or pericarditis were identified retrospectively using the EAVE-II GP dataset. Consultation level data was extracted covering the period 1 September 2020 to19 August 2023. Myocarditis or pericarditis events were identified based on the presence of a corresponding Read Code [**S7**] within the consultation record. Scottish COVID-19 vaccination, GP consultation and mortality were linked using unique identifiers. There was no GP recorded myo/pericarditis in children 5-11 years following vaccination.</td></tr>
<tr><td>8. Statistical packages used</td><td colspan="4">All analyses were conducted in R version 3.6.1, using the tidyverse [**S8**], survival [**S9**], tidylog [**S10**] and lubridate [**S11**] packages.</td></tr>
<tr><td rowspan="3">9. Self-controlled case series (SCCS) results for each health outcome with at least 5 admissions in the risk periods following a dose of BNT162b2 vaccine.</td><td>Health outcome and risk period (days)</td><td>IRR</td><td>95% CI</td><td>p-value</td></tr>
<tr><td>Type 1 diabetes</td><td></td><td></td><td></td></tr>
<tr><td>1–90 days</td><td>0.66</td><td>0.36, 1.23</td><td>0.12</td></tr>
</table>

Table S6. Checklist: the STROBE and RECORD statements.

	Item No.	STROBE items	RECORD items	Location in manuscript where items are reported
**Title and abstract**				
	1	(a) Indicate the study's design with a commonly used term in the title or the abstract (b) Provide in the abstract an informative and balanced summary of what was done and what was found	RECORD 1.1: The type of data used should be specified in the title or abstract. When possible, the name of the databases used should be included.<br><br>RECORD 1.2: If applicable, the geographic region and timeframe within which the study took place should be reported in the title or abstract.<br><br>RECORD 1.3: If linkage between databases was conducted for the study, this should be clearly stated in the title or abstract.	S1a: Abstract methods section & Methods: *"This national prospective cohort study…".*<br>S1b: Provided in the abstract.<br>R1.1: Provided in the abstract methods section (EAVEII)<br>R1.2.: Provided in the abstract results section<br>R1.3.: Provided in the abstract methods section
**Introduction**				
Background rationale	2	Explain the scientific background and rationale for the investigation being reported		S2: Provided in the introduction and Supplementary table S2
Objectives	3	State specific objectives, including any prespecified hypotheses		S3: Provided in the introduction, last para
**Methods**				
Study Design	4	Present key elements of study design early in the paper		S4: Provided in the abstract, methods section; and main paper methods section
Setting	5	Describe the setting, locations, and relevant dates, including periods of recruitment, exposure, follow-up, and data collection		S5: Provided in the methods section, para 1-3.
Participants	6	*(a) Cohort study* - Give the eligibility criteria, and the sources and methods of selection of participants. Describe methods of follow-up<br>*Case-control study* - Give the eligibility criteria, and the sources and methods of case ascertainment and control selection. Give the rationale for the choice of cases and controls<br>*Cross-sectional study* - Give the eligibility criteria, and the sources and methods of selection of participants<br><br>*(b) Cohort study* - For matched studies, give matching criteria and number of exposed and unexposed<br>*Case-control study* - For matched studies, give matching criteria and the number of controls per case	RECORD 6.1: The methods of study population selection (such as codes or algorithms used to identify subjects) should be listed in detail. If this is not possible, an explanation should be provided.<br><br>RECORD 6.2: Any validation studies of the codes or algorithms used to select the population should be referenced. If validation was conducted for this study and not published elsewhere, detailed methods and results should be provided.<br><br>RECORD 6.3: If the study involved linkage of	S6a & R6.1 & R6.2: The data source and linkage process are described in the methods section and Supplementary table S2, including references to validation studies. Supplementary figures 2,3 explain cohorts, while Supplementary figures 4-6 serve to explore possible testing-related biases and validate tests to an extent.<br><br>R6.3. Supplementary figure S1 provides data diagram of primary data sources.

			databases, consider use of a flow diagram or other graphical display to demonstrate the data linkage process, including the number of individuals with linked data at each stage.	
Variables	7	Clearly define all outcomes, exposures, predictors, potential confounders, and effect modifiers. Give diagnostic criteria, if applicable.	RECORD 7.1: A complete list of codes and algorithms used to classify exposures, outcomes, confounders, and effect modifiers should be provided. If these cannot be reported, an explanation should be provided.	S7 & R7.1: These can be found in the Methods section (under Primary data sources) and Supplementary tables S1 and S2.
Data sources/ measurement	8	For each variable of interest, give sources of data and details of methods of assessment (measurement).<br>Describe comparability of assessment methods if there is more than one group		S8: This is provided in all parts of the Methods section and Supplementary tables S1 and S2.
Bias	9	Describe any efforts to address potential sources of bias		S9: Methods section, under "Vaccine safety" and under "Vaccine effectiveness and waning", and supplementary table S2.
Study size	10	Explain how the study size was arrived at		S10: This is a national study (the whole population is included)
Quantitative variables	11	Explain how quantitative variables were handled in the analyses. If applicable, describe which groupings were chosen, and why		S11: "Methods" section, "Primary data sources"; entire sections "Vaccine safety" and "Vaccine effectiveness and waning"; and Supplementary table S2
Statistical methods	12	(a) Describe all statistical methods, including those used to control for confounding<br>(b) Describe any methods used to examine subgroups and interactions<br>(c) Explain how missing data were addressed<br>(d) *Cohort study* - If applicable, explain how loss to follow-up was addressed<br>*Case-control study* - If applicable, explain how matching of cases and controls was addressed<br>*Cross-sectional study* - If applicable, describe analytical methods taking account of sampling strategy<br>(e) Describe any sensitivity analyses		S12: Methods section, under "Vaccine safety", under "Vaccine effectiveness and waning"; and Supplementary table S2;
Data access and cleaning methods		..	RECORD 12.1: Authors should describe the extent to which the investigators had access to the database population used to create the study population.	R12.1.: "Methods" section, "Primary data sources" para 1-2; and Supplementary figure S1; references 45-48.<br>R12.2.: references 45-48

			RECORD 12.2: Authors should provide information on the data cleaning methods used in the study.	
Linkage		..	RECORD 12.3: State whether the study included person-level, institutional-level, or other data linkage across two or more databases. The methods of linkage and methods of linkage quality evaluation should be provided.	R12.3.: "Methods" section, "Primary data sources" para 1-2; and Supplementary figures S1-S6; references 45-48.
**Results**				
Participants	13	(a) Report the numbers of individuals at each stage of the study (*e.g.*, numbers potentially eligible, examined for eligibility, confirmed eligible, included in the study, completing follow-up, and analysed)<br>(b) Give reasons for non-participation at each stage.<br>(c) Consider use of a flow diagram	RECORD 13.1: Describe in detail the selection of the persons included in the study (*i.e.,* study population selection) including filtering based on data quality, data availability and linkage. The selection of included persons can be described in the text and/or by means of the study flow diagram.	S13 & R13.1: "Methods" section, "Primary data sources" para 1-2; and Supplementary figure S1; references 45-48.
Descriptive data	14	(a) Give characteristics of study participants (*e.g.*, demographic, clinical, social) and information on exposures and potential confounders<br>(b) Indicate the number of participants with missing data for each variable of interest<br>(c) *Cohort study* - summarise follow-up time (*e.g.*, average and total amount)		S14: "Results" section, Table 1; Supplementary tables S3-S5;
Outcome data	15	*Cohort study* - Report numbers of outcome events or summary measures over time<br>*Case-control study* - Report numbers in each exposure category, or summary measures of exposure<br>*Cross-sectional study* - Report numbers of outcome events or summary measures		S15: "Results" section, Tables 1-4;
Main results	16	(a) Give unadjusted estimates and, if applicable, confounder-adjusted estimates and their precision (e.g., 95% confidence interval). Make clear which confounders were adjusted for and why they were included<br>(b) Report category boundaries when continuous variables were categorized<br>(c) If relevant, consider translating estimates of relative risk into absolute risk for a meaningful time period		S16: "Results" section, paragraphs 2-6; Figure 2;
Other analyses	17	Report other analyses done—e.g., analyses of subgroups and		PS17: "Results" section, para 3; Supplementary table S2, S5;

		interactions, and sensitivity analyses		
**Discussion**				
Key results	18	Summarise key results with reference to study objectives		S18: “Discussion” section, sub-section “Main findings”
Limitations	19	Discuss limitations of the study, considering sources of potential bias or imprecision. Discuss both direction and magnitude of any potential bias	RECORD 19.1: Discuss the implications of using data that were not created or collected to answer the specific research question(s). Include discussion of misclassification bias, unmeasured confounding, missing data, and changing eligibility over time, as they pertain to the study being reported.	S19, R19.1: “Discussion” section, sub-section on “Limitations of this study”
Interpretation	20	Give a cautious overall interpretation of results considering objectives, limitations, multiplicity of analyses, results from similar studies, and other relevant evidence		S20: “Discussion” section, sub-sections on “Safety analysis” and “Analysis of the Effectiveness and Waning”
Generalisa-bility	21	Discuss the generalisability (external validity) of the study results		S21: “Discussion” section, sub-section on “Implications for policy, practice and research” and Box 1
**Other Information**				
Funding	22	Give the source of funding and the role of the funders for the present study and, if applicable, for the original study on which the present article is based		S22: We included a specific statement about the funding role.
Accessibility of protocol, raw data, and programming code		..	RECORD 22.1: Authors should provide information on how to access any supplemental information such as the study protocol, raw data, or programming code.	R22.1.: We included a specific section on data availability.

References:

S1. Clift AK, Coupland CAC, Keogh RH, Diaz-Ordaz K, Williamson E, Harrison EM, Hayward A, Hemingway H, Horby P, Mehta N, Benger J, Khunti K, Spiegelhalter D, Sheikh A, Valabhji J, Lyons RA, Robson J, Semple MG, Kee F, Johnson P, Jebb S, Williams T, Hippisley-Cox J. Living risk prediction algorithm (QCOVID) for risk of hospital admission and mortality from coronavirus 19 in adults: national derivation and validation cohort study. BMJ. 2020 Oct 20;371:m3731. doi: 10.1136/bmj.m3731. PMID: 33082154; PMCID: PMC7574532.

S2. QCovid™ risk calculator. Available from: https://qcovid.org; Accessed: Mar 31, 2022;

S3.https://assets.publishing.service.gov.uk/government/uploads/system/uploads/attachment_data/file/1057798/Greenbook-chapter-14a-28Feb22.pdf; pp. 18-26, Table 4, Box 1 and Box 2;

S4. https://www.brit-thoracic.org.uk/covid-19/covid-19-information-for-the-respiratory-community/#jcvi-advice-oncovid-19-vaccination-for-children-aged-12-15-years-in-clinical-at-risk-groups)

S5. ISD Scotland | Information Services Division. Available from: https://www.ndc.scot.nhs.uk/Dictionary-A-Z/Definitions/index.asp?Search=E&ID=241&Title=Episode%20of%20Care (Accessed: 26 Mar 2022)

S6. Turas Vaccination Management tool | Turas | Learn. Available from: https://learn.nes.nhs.scot/42708/turas-vaccination-management-tool (Accessed: 26 Mar 2022)

S7. Read Codes - NHS Digital. Available from: https://digital.nhs.uk/services/terminology-and-classifications/read-codes. (Accessed: 26 Mar 2022)

S8. Wickham H. Easily Install and Load the “Tidyverse” [R package tidyverse version 1.3.1]. 2021.

S9. Therneau TM. Survival Analysis [R package survival version 3.3-1]. 2022.

S10. Elbers B. Logging for “dplyr” and “tidyr” Functions [R package tidylog version 1.0.2]. 2020.

S11. Make Dealing with Dates a Little Easier [R package lubridate version 1.8.0]. 7 Oct 2021. Available from: https://cran.r-project.org/web/packages/lubridate/index.html. (Accessed: 26 Mar 2022)

S12. https://scotland.shinyapps.io/phs-covid19-education/_w_852fb58e/
